# Spontaneous symmetry breaking in vortex systems with two repulsive lengthscales

**DOI:** 10.1038/srep15569

**Published:** 2015-10-23

**Authors:** P. J. Curran, W. M. Desoky, M. V. Milos̆ević, A. Chaves, J.-B. Laloë, J. S. Moodera, S. J. Bending

**Affiliations:** 1 University of Bath, Department of Physics, Bath, BA2 7AY, UK; 2 University of Zagazig, Department of Physics, Ash Sharqiyah, Egypt; 3 Universiteit Antwerpen, Departement Fysica, Groenenborgerlaan 171, B-2020 Antwerpen, Belgium; 4 Departamento de Física, Universidade Federal do Ceará, Caixa Postal 6030, Campus do Pici, 60455-900 Fortaleza, Ceará, Brazil; 5 Francis Bitter Magnet Laboratory, MIT, 170 Albany Street, Cambridge, MA 02139, USA

## Abstract

Scanning Hall probe microscopy (SHPM) has been used to study vortex structures in thin epitaxial films of the superconductor MgB_2_. Unusual vortex patterns observed in MgB_2_ single crystals have previously been attributed to a competition between short-range repulsive and long-range attractive vortex-vortex interactions in this two band superconductor; the type 1.5 superconductivity scenario. Our films have much higher levels of disorder than bulk single crystals and therefore *both* superconducting condensates are expected to be pushed deep into the type 2 regime with purely repulsive vortex interactions. We observe broken symmetry vortex patterns at low fields in all samples after field-cooling from above T_c_. These are consistent with those seen in systems with competing repulsions on disparate length scales, and remarkably similar structures are reproduced in dirty two band Ginzburg-Landau calculations, where the simulation parameters have been defined by experimental observations. This suggests that in our dirty MgB_2_ films, the symmetry of the vortex structures is broken by the presence of vortex *repulsions* with two different lengthscales, originating from the two distinct superconducting condensates. This represents an entirely new mechanism for spontaneous symmetry breaking in systems of superconducting vortices, with important implications for pinning phenomena and high current density applications.

The spontaneous breaking of symmetry is a pervasive theme throughout physics and is responsible for many phenomena that occur in a diverse range of systems, ranging from phase transitions in condensed matter to the inequalities of mass of the elementary particles. Some of the most aesthetically pleasing results of symmetry breaking are those observed in systems where spontaneous pattern formation stabilises one of several standard morphologies such as stripes, clumps, bubbles and labyrinths^1^. Patterns with striking similarities are observed in a wide variety of systems of interacting particles such as chemical mixtures (Turing patterns), colloids, convective roll patterns in gases, Cahn-Hilliard systems^2^, and type 1 superconducting thin films, where self-organisation arises from competition between interactions on different lengthscales^3^. Commonly these systems involve a long-range attraction in harness with a short-range repulsion, but interestingly, and somewhat counter-intuitively, here we show that remarkably similar patterns can also be realised by systems of purely ***repulsive*** isotropic interactions with multiple lengthscales^4^,^5^,^6^,^7^,^8^,^9^.

There is great interest in the study of superconducting vortices in type 2 superconductors as a two-dimensional (2D) analog of systems of particles interacting in isotropic potentials^8^,^10^,^11^. Here the particle (vortex) density and interaction strength can be varied over several orders of magnitude by changing the applied magnetic field. In general, vortices in type 2 superconductors interact via a purely monotonic repulsive force but several exceptions are known to exist which result in a non-monotonic interaction^12^. A new and exciting mechanism recently emerged from the vibrant field of multi-band superconductivity, where disparate lengthscales arise from two or more weakly coupled superconducting condensates^13^,^14^,^15^. Bitter decoration and scanning probe measurements on single crystals of the two-band superconductor MgB_2_ have revealed unconventional vortex patterns^16^,^17^,^18^,^19^, consistent with those seen in systems of particles interacting with non-monotonic potentials^20^. Hence, this material appears to be a model system for the investigation of vortex structures interacting over two independent length scales.

An attraction between vortices can arise from the gain in condensation energy derived from the overlap of the normal cores of size *ξ* (coherence length), and a repulsion from the overlap of the vortex supercurrents flowing at a distance *λ* (penetration depth) from the core. It follows that in two-band superconductors the inter-vortex potential, and resultant vortex patterns, are acutely dependent on the superconducting lengthscales (*λ*(*λ*_1_, *λ*_2_) and *ξ*_1_, *ξ*_2_) associated with each band condensate. To date, observations of vortex structures in MgB_2_ have been limited to ultra-pure single crystals which are assumed to be in the clean superconducting limit (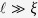) but it is insightful to systematically tune these parameters via the controlled growth of MgB_2_ thin films, whose level of disorder is a function of film thickness. Thin films are in the dirty limit when the electronic mean free path, 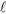, falls below the superconducting coherence length, *ξ*, when the characteristic superconducting length scales become highly dependent on 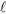. In this way the detail of the vortex-vortex interaction can be systematically tuned. Furthermore, the long-range repulsion of vortices due to the interaction of their magnetic field outside the film becomes important, and adds another degree of tunability to the overall vortex interaction.

## Experimental Method

High resolution scanning Hall probe microscopy (SHPM) has been used to perform local magnetic imaging of two epitaxial MgB_2_ thin-films of thicknesses 77 nm and 160 nm. The films were grown by molecular beam epitaxy on a Silicon (111) substrate held at ≈300 °C at a growth rate of ~0.23 nm/s with a typical flux ratio (Mg:B) of 1.8^21^. A 5 nm MgO seed layer was evaporated onto the clean Si(111) surface to promote lattice matching. A lattice strain as low as 3% is achievable through careful consideration of substrate orientation^21^. Any strain associated with the lattice mismatch resides in the immediate vicinity of the seed layer and is expected to have a negligible effect on MgB_2_ thin films with the thicknesses studied here^22^.

**Figure 1:**
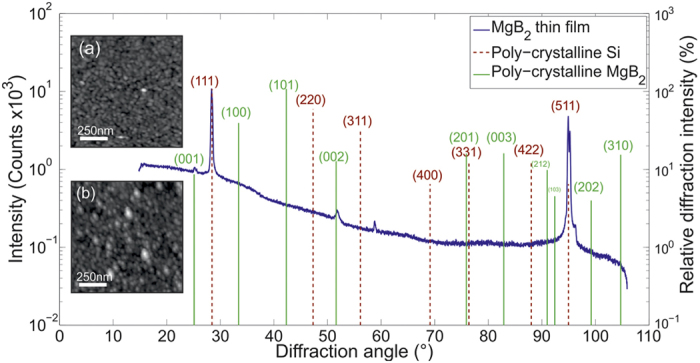
X-ray diffraction data of a 160 nm MgB_2_ thin-film. Vertical lines represent powder diffraction lines of polycrystalline samples. Inset are AFM images of a 77 nm (a) and a 160 nm (b) film.

Figure 1 displays x-ray diffraction (XRD) data for the 160 nm sample which establishes that the film is highly textured. The diffraction peak markers (vertical lines) represent powder diffraction patterns of polycrystalline MgB_2_ samples from standard XRD reference tables and the line height represents the relative diffraction intensity. The large height of the peaks for the (002) and (001) planes, which have a relative powder pattern intensity of <10%, indicates a strong texture in the expected growth orientation on Si(111). This conclusion is further supported by the absence of the MgB_2_ (101) peak which has the maximum powder weighting. The small signal recorded at ≈58.5° is a forbidden reflection from the Si(222) planes, picked up because of the type of 2D detector used.

**Figure 2:**
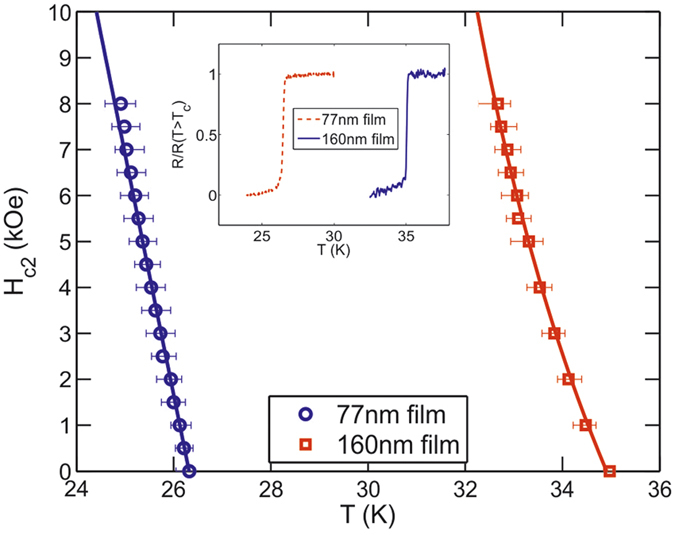
H_c2_(*T*) for the 77 nm and 160 nm films. Solid lines are theoretical fits to the model of Gurevich *et al.*^23^. The normalised sheet resistance as a function of temperature at H = 0 is inset.

Figure 2 shows *H*_c2_(T) data, obtained from electrical transport measurements, which suggests that the two films have strikingly disparate scattering rates. Experimental *H*_c2_(T) data for a number of MgB_2_ thin-films have previously been successfully analysed within a theory of dirty two-band superconductivity due to Gurevich, in which impurity scattering is introduced via intraband electron diffusivities, *D*_*π*_, *D*_*σ*_, and the interband scattering parameter, g^23^. Following the approach of Noguchi *et al.*^24^, we choose the fixed intraband (*λ*_*ππ*_, *λ*_*σσ*_) and interband coupling constants (*λ*_*πσ*_, *λ*_*σπ*_) as 0.285, 0.81, 0.09 and 0.119 respectively, and calculate *g*(*T*_c_) for our films to be *g*_77nm_ = 0.44 and *g*_160nm_ = 0.081, confirming that there are significantly higher levels of scattering in the 77 nm film.

This view is further substantiated by estimates of the electronic mean free path, 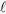, which has been extracted from normalised sheet resistance data (Fig. 2 inset) via the Drude relation 
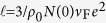
. Using an average Fermi velocity of *ν*_F_ = 4.8 × 10^5^ ms^−1^ 25,26, and a density of states *N*(0) = 0.7 eV^−1^ unit cell^−1^ 27,28, with residual resistivity data *ρ*_77nm_(27 K) = 39.0 ± 0.5 *μ*Ωcm and *ρ*_160nm_(36 K) = 14.88 ± 0.02 *μ*Ωcm we estimate 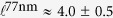 nm and 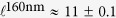 nm in the 77 nm and 160 nm films respectively. As expected these are far shorter than estimates for single crystals in the literature which lie in the range 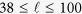 nm^29^,^30^,^31^,^32^,^33^,^34^. The high scattering-rate scenario is best illustrated by the dramatically reduced zero-field critical temperature of the 77 nm film with respect to the 160 nm film and single crystals, (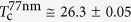 K 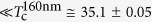 K 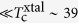 K)^16^, which is a well-known consequence of increased interband scattering in two-band superconductors^23^,^35^,^36^.

SHPM is a quantitative and non-invasive imaging technique for mapping surface magnetic fields^37^. Conventional scanning probe microscopy technology is utilised to scan a sub-micron Hall effect sensor just above the surface of the sample to be imaged in order to generate two-dimensional maps of the local magnetic induction. The Hall probe was fabricated in a GaAs/AlGaAs heterostructure chip. Electron beam lithography and wet chemical etching were used to define a 0.8 *μ*m Hall probe in the two-dimensional electron gas approximately 5 *μ*m from the corner of a deep mesa etch, which was coated with a thin Au layer to act as an integrated scanning tunneling microscope (STM) tip. The Hall probe is mounted on a piezoelectric scanning tube, which itself is mounted on three nanopositioners which allow coarse motion in X-Y-Z. The Hall probe is approached towards the sample until tunnelling is established and then typically retracted ≈200 nm out of tunnel contact, and rapidly scanned across the surface to measure the local magnetic field distribution. Each individual 2D map of the magnetic induction usually comprises 128 × 128 pixels covering a scan area of 14 × 14 *μ*m^2^ at 1.7 K. The spatial resolution is limited by the size of the Hall sensor or the sample/sensor separation, whichever is the greater of the two.

### Data accession

All of the data discussed in this manuscript can be found at http://doi.org/10.15125/BATH-00152.

## Results

**Figure 3:**
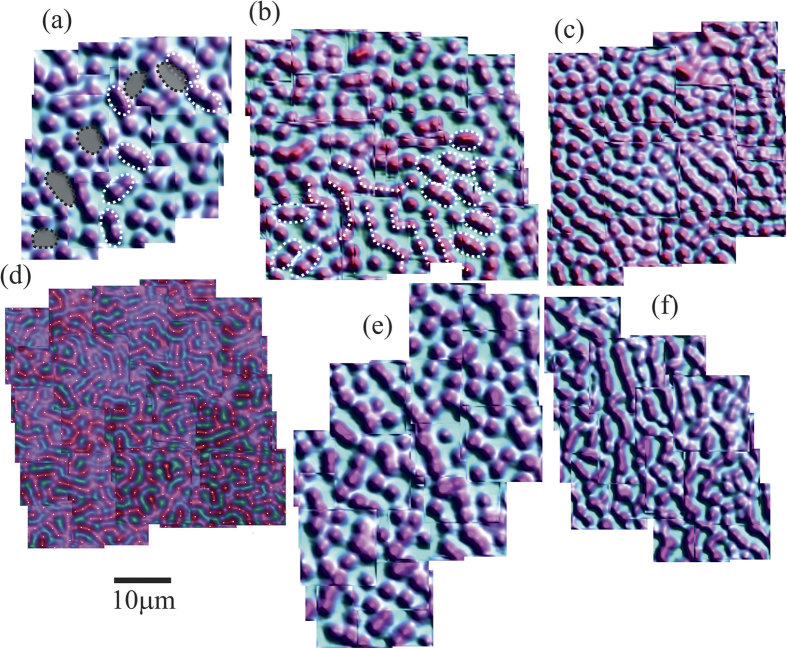
Scanning Hall probe images (≈50 × 50 *μ*m^2^) of the vortex distribution in a 160 nm thick superconducting MgB_2_ film at T = 1.7 K and fields of (a) 1.25 G, (b) 1.7 G, (c) 2.8 G and (d) 5 G, and a 77 nm film at (e) 1.25 G and (f) 2.8 G. Typical examples of dimers and voids are indicated in (**a**), short chains in (**b**) while vortex locations in (**d**) are highlighted with white dots.

**Figure 4:**
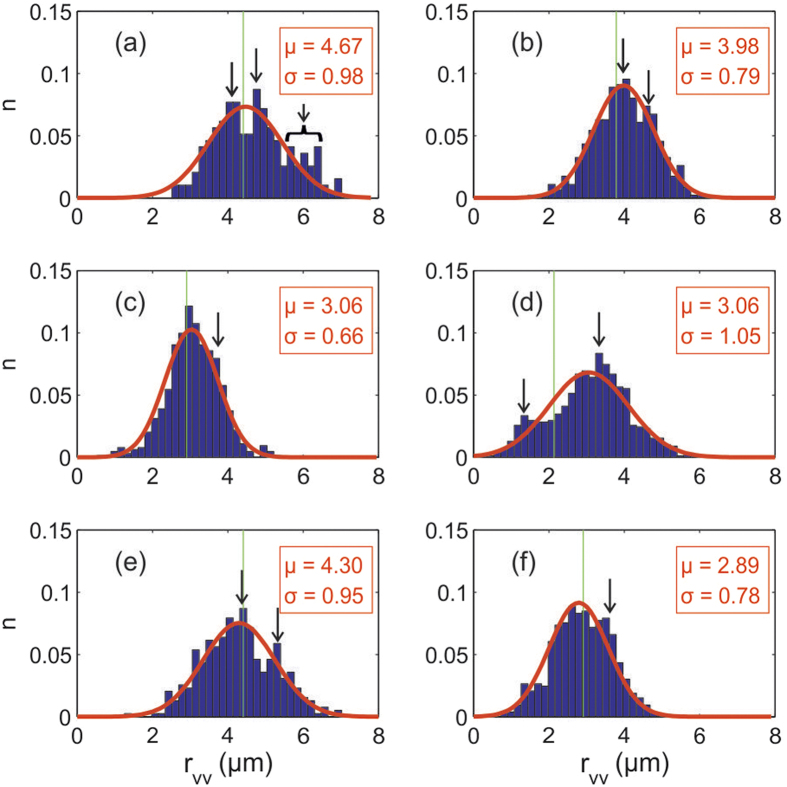
Normalised histograms of the vortex-vortex nearest neighbor distributions of a 160 nm film in applied fields of (a) 1.25 G, (b) 1.75 G, (c) 2.8 G, (d) 5 G, and a 77 nm film in (e) 1.25 G, (f) 2.8 G. The smooth curves are Gaussian fits with the indicated mean and standard deviation. The vortex spacing for an ideal Abrikosov triangular lattice is indicated by the vertical line.

Figure 3 displays the vortex distributions captured in both films after field cooling from *T* > *T*_c_ to *T* ≈ 1.7 K, at four different magnetic inductions (1.25, 1.7, 2.8 and 5 G), with the field perpendicular to the plane of the film. Each image is a composite of either 16 or 25 individual scans, allowing one to assess the degree of the order in the vortex patterns over an area of ≈50 × 50 *μ*m^2^. Shearing in the field of view arises from asymmetry in the movement of the microscope coarse positioners at low temperature. An initial visual inspection of the sequence of fields in the 160 nm film, (a) to (d), shows symmetry breaking at all fields where the predominant vortex patterns can be classed as an evolution from: dimers, coexistence of dimers and chains, chains with a predominant stripe direction, and finally a labyrinth-like structure at the highest field. The intermediate fields studied in the 77 nm film, (e) and (f), also show chains with a predominant direction. Figure 4 presents normalised histograms of the nearest neighbour vortex-vortex separations based on Delaunay triangulation of the images, which is a useful statistical technique for characterising vortex clustering and other types of symmetry breaking.

Considering first the evolution of the vortex distributions in the 160 nm film, (a) to (d), at the lowest field (1.25 G) we see a weakly split bi-modal distribution, spread around the ideal triangular spacing *a*_*tri*_(1.25 *G*) = (*ϕ*_0_/sin(60).*B*)^1/2^ (vertical line). The two peaks reflect the average intra- and inter-chain vortex separations. The weak broad shoulder at higher bond lengths (~6 *μ*m) reflects the small number of voids in the vortex distribution. At 1.7 G the peak splitting is weaker, the distribution closer to Gaussian and the long distance shoulder has disappeared, reflecting the fact that there are no obvious voids in the vortex distribution at this density. At 2.8 G, peak splitting is no longer observed but a weak long bond shoulder remains, as indicated by the black arrow. At 5 G a rather pronounced splitting occurs, coinciding with the formation of a labyrinth structure. The statistics for the intermediate fields studied in the 77 nm film show similar features to the 160 nm sample, with a clear peak splitting at 1.25 G and a more Gaussian-like distribution, with a weak long-bond shoulder (black arrow) at 2.8 G.

**Figure 5:**
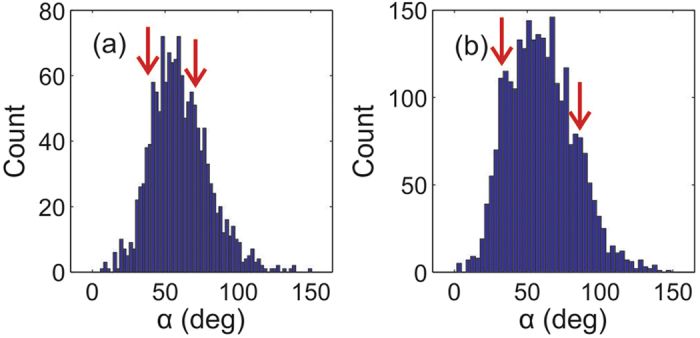
Histograms of the internal angles (*α*) of the Delaunay triangulation of the vortex distributions in the 160 nm film at (a) 2.8 G and (b) 5 G. The small and large angle peaks (arrows) characterise the degree of chaining in the images and show a pronounced divergence from 47° and 68° at 2.8 G to 32° and 88° after the transition from a chain-like state to a labyrinth-like structure at 5 G.

The transition to the labyrinth state from an ordered chain state appears to be accompanied by a dramatic change of the internal angles of the Delaunay triangulation (*α*). The *α* histogram of a weakly disordered triangular lattice will have a gaussian distribution around a mean angle of 60°. Lower symmetry chaining states can be viewed as the stretching or squashing of the hexagonal lattice and are characterised by the appearance of secondary peaks at small (*α* < 60°) and large (*α* > 60°) angles in the *α* histogram. Figure 5 shows the distribution of *α* from the vortex patterns in the 160 nm film at 2.8 G (a) and 5 G (b). These images show the highest degree of order and are at fields directly before and after the onset of a labyrinth-like structure. They illustrate the marked difference in the upper and lower modes (red arrows). In particular, the labyrinth-like state is characterised by a higher fraction of large bond angles, close to 90°, that reflect the distortion of chains into zig-zag patterns.

## Discussion

It is well established that vortex pinning at material defects can break the symmetry of the vortex lattice in type 2 superconductors^38^. It is therefore important to rule out the influence of defects on the vortex structures observed here, in particular since studies of critical currents in MgB_2_ thin-films suggest that grain boundaries are the dominant pinning sites in this system^39^. Atomic force microscopy images of these epitaxially grown films (c.f., Fig. 1 inset) show the grain size to be ~10–75 nm in the 77 nm film, and ~50–100 nm in the 160 nm film which is much too fine to correlate with the vortex patterns observed here. Secondly, the characteristic direction of vortex chains varies at each field, including several instances of curved chains. Hence they seem unlikely to have arisen from crystallographic defects with a fixed linear geometry.

**Figure 6:**
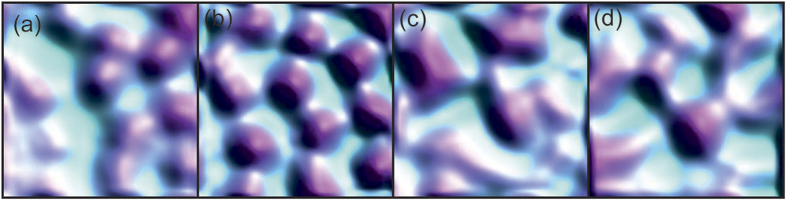
A sequence of repeated field-cooled cycles in applied fields of 2.5 G, (a,b), and 5 G, (c,d). The images were acquired after cooling to 20 K. The field of view is ≈8 × 8 *μ*m^2^.

To gain further insights into the role of pinning we have analysed vortex structures after repeated field-cooled (FC) cycles at the same location, such as those shown in Fig. 6. At 2.5 G, (a) and (b), the images are qualitatively similar, albeit with small shifts in vortex location. This tendency for vortex chains/stripes to occupy the same location and orientation on repeated field-cooling in low fields was also observed in SHPM studies of high-quality single crystals^19^. At 5 G however, the vortex distribution is completely different after repeated FC cycles which definitively rules out any role for the underlying pinning potential in determining the vortex distribution at this vortex density.

We now address the question as to what is causing the symmetry of the vortex distributions to be broken in this way. Smectic phases of orientated stripes are known to form in the presence of shear forces^40^,^41^, and local shear forces could arise in this system if a critical percentage of vortices was strongly pinned. However, our field-cooled patterns should closely approximate an equilibrium distribution and the presence of long-range shear forces seems highly unlikely. Vortex chains have also been shown to form in anisotropic superconductors when a component of the applied field lies in the sample plane, or due to the interaction with supercurrents flowing at the sample edges. However, care was taken to minimise the in-plane field component during experiments and images were captured ~1 mm from the sample perimeter, allowing us to exclude these possibilities.

To simulate the observed vortex behavior we employ the dirty limit two-gap Ginzburg-Landau (GL) formalism^36^, where interband scattering and modified diffusivities introduce new effects to the standard clean limit formulation^42^. In the dirty case, the vortex behavior is governed by the GL free energy ∫*FdV*, with


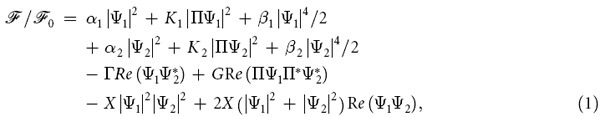


where Π = ∇ + 2*πiA*/*ϕ*_0_, *A* is the magnetic vector potential and *ϕ*_0_ is the flux quantum. The GL expansion coefficients are given by


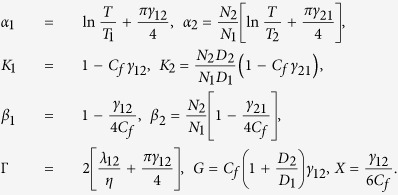


Here *N*_*j*__ = 1,2_ are the band densities of states, and *D*_*j*_ _ =1,2_ are the corresponding electron diffusivities. *T*_1_ = *T*_*c*0_ exp[−(*λ*_0_ − *λ*_−_)/2*η*], and *T*_2_ = *T*_*c*0_ exp[−(*λ*_0_ + *λ*_−_)/2*η*], with 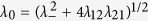, and *λ*_−_ = *λ*_11_ − *λ*_22_, and *η* the determinant of the BCS coupling matrix 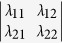. Compared to the notation in the Gurevich theory^36^, our interband scattering rates *γ*_*mm*′_ are scaled by *T*_*c*_, and are related to the interband scattering parameter as 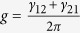. Furthermore, the free energy of Eq. (1) is expressed in units of 
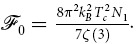
. The constant *C*_*f*_ featuring in several coefficients is *C*_*f*_ = 14*ζ*(3)/*π*^3^ (*ζ*(3) stands for Riemann zeta function), and finally, all distances are scaled to 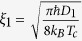.

In what follows, we use the parameters as estimated from the experiment, i.e. the bulk MgB_2_ values for the coupling constants and the densities of states, but the electron diffusivities (*D*_*σ*_ = 0.23 cm^2^s^−1^, *D*_*π*_ = 40 cm^2^s^−1^), *T*_*c*_( = 35.9 K), and interband scattering rates (*γ*_12_ = 0.2877- and *γ*_21_ = 0.2212) from the fit of the measured H_c2(*T*)_ for the 160 nm thick MgB_2_ film. With these parameters, the nominal lengthscales of the condensates forming in the *σ* and *π* (at T = 0) bands become


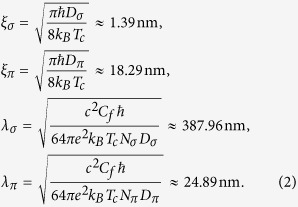


This sets both band condensates in the type 2 regime, with very disparate penetration depths. In our 160 nm thick film, *λ*_*σ*_ ≫ *d* and so the effective penetration depth will be further enhanced, approaching 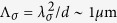, (d is film thickness) whereas *λ*_*π*_ ≪ *d* and will therefore be unaffected by film thickness. Notably, the penetration depths define the decay of vortical supercurrents in each condensate, and thereby affect the vortex-vortex interaction and resultant pattern formation. In thin single-band films, one finds the vortex interaction:^43^


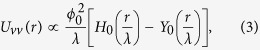


where *H*_0_ is the Struve function, and *Y*_0_ stands for the Bessel function of the second kind. To a first approximation, the vortex-vortex interaction will be repulsive and in our case can be seen as a superposition of the vortex interactions in two condensates. It therefore will be repulsive with two lengthscales - a short-range repulsion linked to the *π* condensate, and long-range one mainly determined by the *σ* condensate. This will remain the case even for moderately strong interband coupling, since the contribution of the *σ* band to the vortex interaction is always very weak at short distances and vice versa (due to the division by *λ*_*j*_ in equation 3). However, the coupling between the condensates has a very nontrivial effect on the vortex interaction, since it couples not only Cooper-pair densities but also resulting supercurrents in two condensates, all of which have different length scales (in the present case, *ξ*_*sigma*_ < *ξ*_*pi*_ < *λ*_*pi*_ < *λ*_*sigma*_). All together, we have the necessary conditions to expect unconventional vortex patterns to appear, such as those predicted to occur in systems of classical particles interacting via repulsive hard and soft potentials with two distinct length scales^4^, which bear a striking resemblance to the vortex patterns we observe here. Similar phenomenology has been discussed in the context of bi- or multilayer superconductors^9^, but we show for the first time the consequences of multiscale repulsion in a film, due to specific properties of the material, not a heterostructure

**Figure 7:**
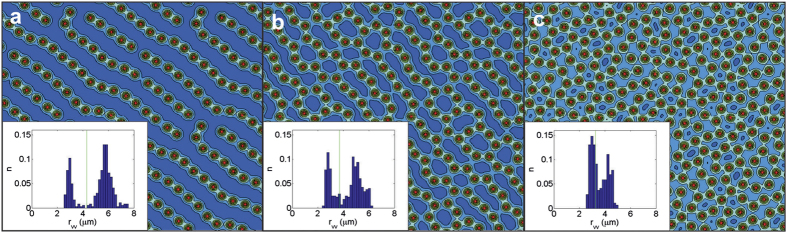
Calculated vortex configurations in a 160 nm thick MgB_2_ film (magnetic field distribution at 500 nm above the surface is shown), for parameters used to fit the experimentally measured *H*_c2_(*T*), and applied magnetic field (a) 1.25 G (size 52.47 × 60.59 *μ*m^2^), (b) 1.7 G (size 51.95 × 60 *μ*m^2^), and (c) 2.2 G (size of the image 51.06 × 58.96 *μ*m^2^). Normalised histograms of the vortex-vortex separations are inset.

To confirm this prediction, we launched a massively parallelized simulation to minimize the functional (1), and compare the results to those obtained in experiment. To make a meaningful comparison, we used an approximately 50 *μ*m × 60 *μ*m simulation region, in the 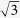-to-1 ratio which should favour formation of the Abrikosov lattice (subject to periodic boundary conditions and the chosen number of flux quanta in the simulation cell). Due to the very small value of *ξ*_*σ*_ ~ 1 *nm*, a valid simulation required a minimum of 100000^2^ grid points, which is an extremely demanding computational task. The results are shown in Fig. 7, as contourplots of the stray magnetic field above the film at applied magnetic fields chosen to match the experimental ones, and with the size of the simulation region adjusted to provide an integer number of vortices in the simulation cell (as required by the virial theorem^44^). We observe clear signatures of the repulsion with two lengthscales, including the formation of vortex chains at the lowest magnetic field, followed by formation of labyrinthal vortex structures and their further evolution towards a disordered vortex lattice at higher fields. The vortex nearest neighbour distributions (inset) are qualitatively similar but more extreme versions of the experimental ones, showing clear bi-modal distributions. Our theoretical simulations reach the predominantly triangular lattice at an applied field of 2.5 G, which is lower than in experiment. Nevertheless, the main features of the vortex patterns, vortex distances within the chains and in-between them, as well as their overall evolution, nicely corroborate the experimental findings. However, the exact explanation of the correlation distances in the observed vortex patterns remains an open question, due to the nontrivial influence of the interband coupling, the proximity of the considered temperature (1.7 K) to the hidden criticality (≈2.3 K^45^,) as well as the known multi-body effects in many vortex physics^46^,^47^,^48^.

Other mechanisms for breaking the symmetry of vortex structures in *single-gap* superconductors are known to exist and for completeness, we now briefly assess their relevance in the context of our results in MgB_2_. Firstly, the stray fields outside the superconductor are known to influence the structure of normal domains in type 1 materials^49^, and vortex patterns in low-*κ* type 2 superconductors^50^. In this mechanism, minimisation of the magnetostatic energy effectively requires the largest mean flux line spacing, resulting in additional vortex repulsion, even in the case of one-band superconductivity. To first approximation the interactions due to the stray fields are already taken into account in the thin film geometry used for our GL simulations. However, this mechanism has been shown at most to lead to the formation of a square vortex lattice in type 1 single-band films^51^, it has never been shown to lead to structures with lower symmetries, e.g., the stripe-like formations observed here. Secondly, a non-monotonic vortex interaction is known to exist in single-band type II/1 materials (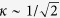), such as high purity Nb where stable vortex patterns of various morphologies have been observed^12^. However, due to the high level of disorder our films are far from this regime, and at any rate the flux filled regions of a type-II/1 superconductor exhibit triangular vortex ordering, which is inconsistent with the stripes/chains observed in our thin films. Therefore we conclude that the presence of two condensates remains essential for the observed behaviour in our thin films.

## Conclusion

We have directly imaged the local stray magnetic fields at the surface of thin films of the two-band superconductor MgB_2_, as a function of applied field. The level of crystalline disorder in our samples is such that we believe *both* bands to be pushed well into the type 2 regime, and crucially, host drastically different penetration depths giving rise to two competing but non-trivially coupled vortex repulsions. We observe an evolution of vortex patterns that bear a striking resemblance to those formed in systems of particles with two repulsions operating on different characteristic length scales, and remarkably similar patterns can be reliably reproduced by dirty two band GL simulations parameterised by our experimental observations. We conclude that the broken symmetry vortex structures observed in MgB_2_ thin films are more than likely to be due to two vortex *repulsions* originating from two distinct supercondcuting condensates which host disparate superconducting length scales.

## Additional Information

**How to cite this article**: Curran, P. J. *et al.* Spontaneous symmetry breaking in vortex systems with two repulsive lengthscales. *Sci. Rep.* **5**, 15569; doi: 10.1038/srep15569 (2015).
